# Development of an Efficient Electroporation Method for Iturin A-Producing *Bacillus subtilis* ZK

**DOI:** 10.3390/ijms16047334

**Published:** 2015-04-01

**Authors:** Zhi Zhang, Zhong-Tao Ding, Dan Shu, Di Luo, Hong Tan

**Affiliations:** 1 Key Laboratory of Environmental and Applied Microbiology, Chengdu Institute of Biology, the Chinese Academy of Sciences, No. 9 Section 4, Renmin Nan Road, Chengdu 610041, China; E-Mails: zhangzhi_asia@163.com (Z.Z.); dingzhongtaochina@126.com (Z.-T.D.); whosecats@163.com (D.S.); lddd24@163.com (D.L.); 2 University of the Chinese Academy of Sciences, No. 19A Yuquan Road, Beijing 100049, China

**Keywords:** electroporation, response surface methodology, wall-weakening agent, wild-type *B. subtilis*, *rapC*

## Abstract

In order to efficiently introduce DNA into *B. subtilis* ZK, which produces iturin A at a high level, we optimized seven electroporation conditions and explored an efficient electroporation method. Using the optimal conditions, the electroporation efficiency was improved to 1.03 × 10^7^ transformants/μg of DNA, an approximately 10,000-fold increase in electroporation efficiency. This efficiency is the highest electroporation efficiency for *B. subtilis* and enables the construction of a directed evolution library or the knockout of a gene in *B. subtilis* ZK for molecular genetics studies. In the optimization process, the combined effects of three types of wall-weakening agents were evaluated using a response surface methodology (RSM) design, which led to a two orders of magnitude increase in electroporation efficiency. To the best of our limited knowledge, this study provides the first demonstration of using an RSM design for optimization of the electroporation conditions for *B. subtilis*. To validate the electroporation efficiency, a case study was performed and a gene (*rapC*) was inactivated in *B. subtilis* ZK using a suicide plasmid pMUTIN4. Moreover, we found that the *rapC* mutants exhibited a marked decrease in iturin A production, suggesting that the *rapC* gene was closely related to the iturin A production.

## 1. Introduction

*B. subtilis*, a type of Gram-positive bacterium, has been used as a cell factory for many enzymes, including proteases, amylases, and lipases [1,2,3]. Some subspecies produce antifungal lipopeptides (surfactin, iturin, and fengycin) that are widely applied for the control of plant pathogenic fungi in agriculture. *B. subtilis* ZK, a wild-type *B. subtilis* with high economic value, has been a workhorse for producing iturin A at a high level [4]. In order to study the high yield mechanism and further improve the iturin A production, it is desirable to perform molecular genetics studies in *B. subtilis* ZK. However, the fact that the genetic manipulation systems of *B. subtilis* ZK, such as the transformation system, gene deletion and mutation library, have not been established retards molecular genetic studies of this organism. The present work aimed at developing an efficient transformation method for the *B. subtilis* ZK in order to carry out molecular genetics studies.

There are five transformation systems available that have been developed to introduce exogenous DNA into *B. subtilis*, such as phage transduction [5], protoplast [6], natural transformation [7], recombinant method [8], and electroporation [9]. The first two methods are important methods for introducing DNA into *Bacillus* [10]. Unfortunately, these two methods have not been wildly used mainly due to heavy workload and time-consuming experimental processes [11]. Natural transformation and recombinant methods are usually used for introducing DNA into *B. subtilis* and lead to high transformation efficiency for some *B. subtilis* [7,8]. However, the transformation efficiency of these two methods is closely related to some genes, such as quorum sensing components, and competence master regulator *comK*. The divergent structures of those genes lead to a huge difference in the transformation efficiency [12,13]. Hence, the natural transformation and recombinant method are strain-specific. In contrast to other methods, electroporation is a common, time-saving transformation method and has been widely used for *Bacillus* [9,10,11,14,15]. Although the electroporation system for *B. subtilis* has been developed for many years, there is still no standard and universal protocol for all *B. subtilis*. We carried out an efficient protocol according to the reported literature [9]. Unfortunately, relatively low efficiency was obtained (about 1.0 × 10^3^ transformants/μg DNA) in wild-type *B. subtilis* ZK and the knockout of a gene was not to be performed, because the electroporation efficiency (1.0 × 10^3^ transformants/μg DNA) was insufficient and the frequency of a foreign plasmid integrating into genome was about 1.0 × 10^−^^4^–1.0 × 10^−6^ per plasmid when the homologous region was less than 1 kb [16,17,18,19].

Many factors affect electroporation efficiency including growth medium [11], growth phase [14], electric field [20], weakening agent [21], plasmid quantity [22], plasmid desalting [23], electroporation buffer [20], and heat treatment [24]. Some factors have combined effects on electroporation efficiency [19,25]. Hence, it is desirable to apply the multifactorial experimental design to investigate the combined effects of the conditions in order to improve electroporation efficiency. Response surface methodology (RSM) is a type of simple, commonly used multifactorial experimental design methodology used for the optimization of a small number of factors. It not only reflects the combined effects of the studied factors, but also allows completion of the optimization through a small number of experiments. To date, RSM design has been widely applied for the optimization of production conditions and media in industrial formulations [26,27] and has been rarely used to optimize electroporation conditions.

In the present study, the effects of seven factors on electroporation were investigated, including the growth media, growth phase, electric field, concentration of weakening agent, electroporation buffer, plasmid quantity, as well as heat treatment. In addition, RSM design was applied to investigate the combined effects of wall-weakening agents on the electroporation efficiency. We ultimately developed an efficient electroporation method for *B. subtilis* ZK that yielded a high efficiency (1.03 × 10^7^ transformants/μg of DNA), which would enable the construction of a large mutant library or the knockout of a gene in wild-type *B. subtilis* ZK. In order to validate the electroporation efficiency, a gene (*rapC*) was successfully inactivated using the suicide plasmid pMUTIN4 with the established electroporation method.

## 2. Results and Discussion

### 2.1. Screening of the Growth Medium for Electro-Competent Cells

An appropriate growth medium is an important factor for determining the electroporation efficiency of bacteria [11,28,29]. In the selected optimal growth medium, four types of growth medium (LBS, LBSP, NCM and BHIS) were investigated to prepare the electro-competent cells of *B. subtilis* ZK. These mediums have been used as the growth mediums for preparing electro-competent in Gram-positive strains [9,11,28]. The first three mediums are suitable for preparing electro-competent cells of *B. amyloliquefaciens*. Nutrient BHIS medium is regarded as the proper medium for the Gram-positve *Corynebacterium glutamicum* [28]. Table 1 shows that the LBSP medium yielded a higher efficiency (1.3 × 10^4^ transformants/μg of DNA) than the LBS, NCM, and BHIS media. Consequently, the LBSP medium was proved to be the appropriate medium for *B. subtilis* ZK and used to prepare the electro-competent cells in the subsequent experiments.

**Table 1:** Effect of growth media on the electroporation efficiency of *B. subtilis* ZK.

Media	EE ^a^	Reference
LBS	4 × 10^3^ ± 0.62 × 10^3^	[9]
LBSP	1.3 × 10^4^ ± 0.17 × 10^4^	[29]
NCM	7 × 10^2^ ± 0.39 × 10^2^	[30]
BHIS	1.6 × 10^2^ ± 0.82 × 10^2^	[31]

When the OD_600_ value reached 0.65, the electro-transformed competent cells were prepared. Then, one hundred nanograms of pHT43 plasmid were used for each electroporation experiment. The field strength was 20 kV∙cm^−1^ and the electroporation buffer was TSM. ^a^ EE, electroporation efficiency (cfu/μg of DNA).

In the previous report, NCM medium yielded higher electroporation efficiency than LBSP medium in *B. amyloliquefaciens* [11]. However, the opposite result was found in the *B. subtilis* ZK and we found some lysis of *B. subtilis* ZK in the NCM medium, which indicated that the ion concentration in NCM may be too high for *B. subtilis* ZK.

### 2.2. Effect of Growth Phase on the Electroporation Efficiency

**Figure 1:**
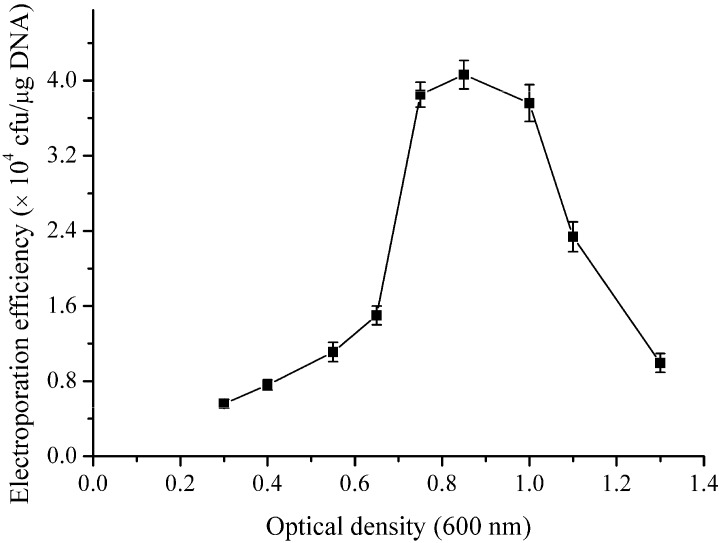
Effects of growth phase of *B. subtilis* ZK on the electroporation efficiency. *B. subtilis* ZK was grown in LBSP medium and the electro-competent cells were prepared at different OD_600_ values (0.3–1.3). One hundred nanograms of the pHT43 plasmid were used for each electroporation experiment. The field strength was 20 kV·cm^−1^ and the electroporation buffer was TSM. The experiments were repeated three times. The error bars indicate the standard deviations from the average values.

Growth phase markedly affects the electroporation efficiency [15,32]. To investigate the effect of growth phase on the electroporation efficiency, electro-competent cells were prepared at different OD_600_ values (0.3–1.3). Figure 1 presents the relationship between the electroporation efficiency and the growth phase. The results show that proper growth phase (OD_600_ 0.7–1) yielded higher electroporation efficiency and an efficiency of 4 × 10^4^ transformants/μg of DNA was obtained when the OD_600_ value reached 0.85. In contrast with results of *B. cereus* [15] and *B. thuringiensis* [32], where the early phase of exponential growth (OD_600_ 0.1–0.4) yielded the highest electroporation efficiency, our results indicated that the late phase of exponential growth (OD_600_ 0.7–1) yielded the highest electroporation efficiency in *B. subtilis* ZK. Similar results have been observed in *B. subtilis* IH6140 and *B. amyloliqueficence* [9], as well as *C. pseudotuberculosis* [22] in which the late phase of exponential growth was required to obtain the highest electroporation efficiency.

### 2.3. Effect of Electric Field on the Electroporation Efficiency

**Figure 2:**
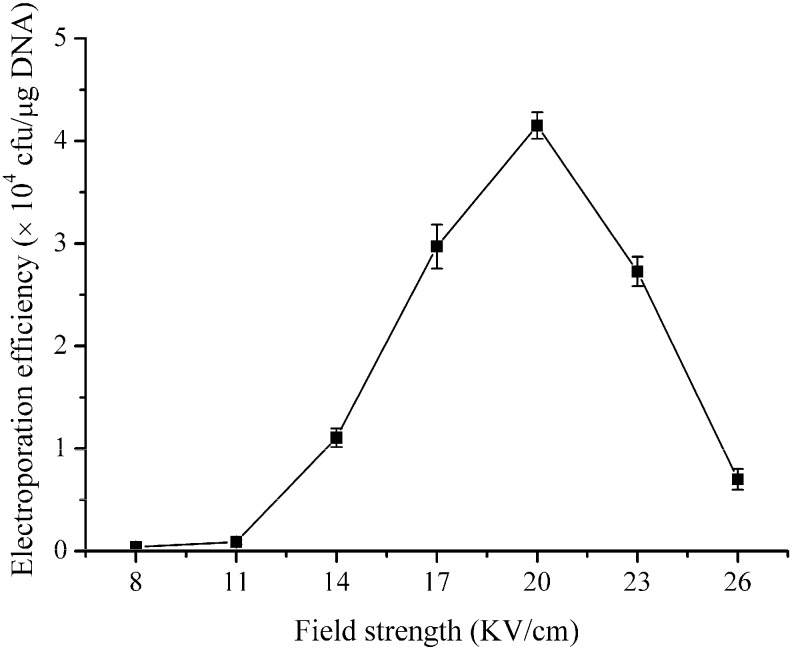
Effects of the field strength on the electroporation efficiency of *B. subtilis* ZK. *B. subtilis* ZK was grown in LBSP medium and the electro-competent cells were prepared when the OD_600_ value reached 0.85. The electroporation experiments were performed under a gradient of the field strength (8–26 kV·cm^−1^). One hundred nanograms of pHT43 plasmid were used for each electroporation experiment, and the electroporation buffer was TSM. The data shown are the averages of three independent experiments, and the error bars indicate the standard deviations from the average values.

Applying high electric field is an approach to overcome the wall of *B. subtilis*, which improves the permeabilization of membrane and produces *Bacillus* cells accessible by exogenous plasmid [11]. However, an exorbitant electric field results in death of cells. To optimize the electric field for *B. subtilis* ZK, electroporation experiments were performed under a gradient of field strength (8–26 kV·cm^−1^). The relationship between the field strength and the electroporation efficiency is shown in Figure 2. An electric field of 20 kV·cm^−1^ led to the optimal efficiency (4 × 10^4^ transformants/μg of DNA), and this value was thus used in the follow-up experiments. The electric fields at around 5–10 kV·cm^−1^ are appropriate conditions for most bacterial cells and can produce the pores accessible by plasmid [10]. In the present study, we found that a high electric field (20 kV·cm^−1^) yielded the highest electroporation efficiency in *B. subtilis* ZK. Similarly high electric field (18–20 kV·cm^−1^) has been observed in *B. amyloliqueficence* [11], *B. cereus* [15], *B. licheniformis* [9], and *B. thuringiensis* [14]. These results indicated that the high electric field was required to achieve the high electroporation efficiency in *Bacillus* strains.

### 2.4. Investigation the Concentration of Wall-Weakening Agent for RSM Design

Application of weakening agents is an important means to improve the electroporation efficiency in *Bacillus*. Glycine, dl-threonine, Tween 80, and ampicillin are widely used to increase the electroporation efficiency in *Bacillus* strains. Glycine and dl-threonine can be integrated into the walls of cells and replace the alanine, deducing the peptidoglycan linkage and loosening the walls of cells [33]. The Tween 80 can increase the electroporation efficiency, likely because the Tween 80 loosens the cell walls and disturbs the cell–membrane fluidity [11].

**Figure 3:**
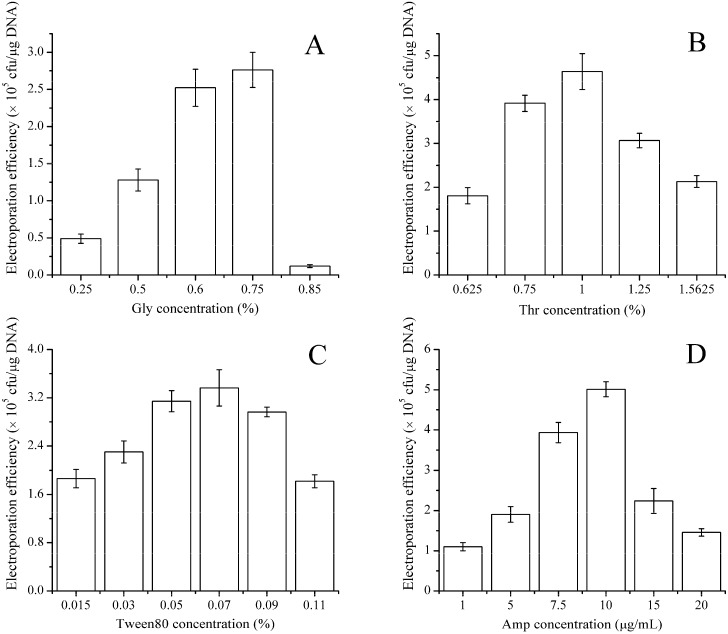
Effects of wall-weakening agents on the electroporation efficiency of *B. subtilis* ZK. Various weakening agents at different concentration gradients (0.25%–0.85% glycine (**A**); 0.63%–1.56% dl-threonine (**B**); 15–110 mg/mL Tween 80 (**C**) and 1–20 μg/mL ampicillin (**D**)) were separately added to the LBSP medium when the OD_600_ value reached 0.5. After shaking for an additional 1 h, the electro-competent cells were prepared. One hundred nanograms of pHT43 were used for each electroporation experiment. The field strength was 20 kV·cm^−1^ and the electroporation buffer was TSM. The values shown are the averages from three independent experiments and the error bars indicate the standard deviations from the average values.

To investigate the concentration of wall-weakening agent for RSM design, the four types of wall-weakening agents (glycine, dl-threonine, Tween 80, and ampicillin) were individually used to prepare the electro-competent cells. The results, which are exhibited in Figure 3, show the individual effect of the weakening agent on the electroporation efficiency. The four types of weakening agents all exhibited an enhancing effect on the electroporation efficiency. Specifically, 0.75% glycine, 1% dl-threonine, and 0.07% Tween 80 yielded efficiencies of 2.75 × 10^5^, 4.5 × 10^5^ and 3.4 × 10^5^ transformants/µg of DNA, respectively (Figure 3A–C). In addition, 10 µg/mL ampicillin showed best effect on the electroporation efficiency and improved the efficiency to 5 × 10^5^ transformants/µg of DNA, which is a 12.5-fold increase compared with the LBSP medium alone (Figure 3D). All these weakening agents yielded significant increases in electroporation efficiency (one order of magnitude) and the order in which the weakening agents contributed to electroporation efficiency was ampicillin > dl-threonine > Tween 80 > glycine. In contrast with the result of *B. cereus* [15], where the weakening agents (glycine, dl-threonine) had a negative effect on the electroporation efficiency, our results indicated that the wall-weakening agents played a crucial role in improving electroporation efficiency for *B. subtilis* ZK. A similar positive effect has been found in *B. amyloliquefaciens* [11], *Gordonia* [34], *B. thuringiensis* [14], and *C. pseudotuberculosis* [22], in which a certain ratio of weakening agents was required to obtain high electroporation efficiency.

### 2.5. RSM Design for Investigating the Combination of Wall-Weakening Agents

Operating parameters have combined effect on the electroporation efficiency [19,25]. To evaluate the combinatorial effects of wall-weakening agents on the electroporation efficiency, a Box-Behnken response surface design (BBD) including 17 trials was used. The BBD experimental design with the predicted and observed electroporation efficiencies are shown in Table 2. The observed electroporation efficiency was regressed to obtain a mathematical model shown in Equation (1). Although ampicillin yielded a 12.5-fold increase in above experiments, it did not exhibit a combined effect with other wall-weakening agents and reduced the electroporation efficiency in combinatorial experiments. Therefore, ampicillin was not used in the combinatorial experiments.


*Y* = 7.3 × 10^6^ − 6.505 × 10^5^·*X*_1_ + 5.633 × 10^5^·*X*_2_ + 7.764 × 10^5^·*X*_3_ + 5.75 × 10^5^·*X*_1_*X*_2_ + 8.141 × 10^5^·*X*_2_*X*_3_ + 8.154 × 10^5^·*X*_1_*X*_3_ − 2.668 × 10^6^·*X*_1_^2^ − 1.144 × 10^7^·*X*_2_ − 3.976 × 10^6^·*X*_3_^2^ (1)

**Table 2:** Box-Behnken response surface (BBD) design with the predicted and observed electroporation efficiencies.

No.	Variables			Electroporation Efficiency (cfu/μg of DNA)	
	*X*_1_	*X*_2_	*X*_3_	Observed	Predicted
1	−1	−1	0	2.1 × 10^6^	2.13 × 10^6^
2	0	0	0	7.28 × 10^6^	7.28 × 10^6^
3	0	0	0	7.2 × 10^6^	7.28 × 10^6^
4	0	−1	−1	2.11 × 10^6^	2.15 × 10^6^
5	0	0	0	7.3 × 10^6^	7.28 × 10^6^
6	1	−1	0	7.1 × 10^5^	6.18 × 10^5^
7	0	0	0	7.28 × 10^6^	7.28 × 10^6^
8	−1	1	0	6.35 × 10^5^	7.14 × 10^5^
9	−1	0	1	1.32 × 10^6^	1.27 × 10^6^
10	0	−1	1	5.28 × 10^5^	5.51 × 10^5^
11	−1	0	−1	3.6 × 10^6^	3.54 × 10^6^
12	0	1	1	5.3 × 10^5^	4.92 × 10^5^
13	0	1	−1	5.8 × 10^5^	5.59 × 10^5^
14	1	0	−1	1.11 × 10^6^	1.15 × 10^6^
15	0	0	0	7.31 × 10^6^	7.28 × 10^6^
16	1	0	1	1.53 × 10^6^	1.6 × 10^6^
17	1	1	0	5.14 × 10^5^	4.94 × 10^5^

When the OD_600_ value reached 0.5, the combined weakening agents were added to the LBSP medium. After shaking for 1 h, the electro-competent competent cells were prepared. Then, one hundred nanograms of pHT43 plasmid were used for each electroporation experiment. The field strength was 20 kV∙cm^−1^ and the electroporation buffer was TSM. *X*_1_: code of glycine, *X*_2_: code of dl-threonine, *X*_3_: code of Tween 80.

A Pareto analysis of variance (ANOVA) was applied to evaluate the adjusted determination coefficient (*R*^2^_Adj_) and the coefficient of variance (CV), which represents the fitness of the quadratic polynomial and the accuracy of the quadratic polynomial, respectively. The *R*^2^_Adj_ of the quadratic polynomial, which reached 0.997, showed good agreement between the predicted and the observed values, indicating that the mathematical model was reliable. The relatively low CV value for the quadratic polynomial (2.5%) exhibited the high precision and reliability of the conducted trials. The ANOVA of the quadratic polynomial is shown in Table 3. The Prob > *F* values of the coefficients are shown in Table 4 and all the coefficients were significant at the desired confidence level.

**Table 3:** ANOVA of the quadratic polynomial.

Source	SS ^a^	DF ^b^	MS ^c^	*F* Value	*p*-Value
Model	1.37 × 10^14^	9	1.522 × 10^13^	2633.10	<0.0001
Residual	4.046 × 10^10^	7	5.78 × 10^9^	-	-
Lack of fit	3.233 × 10^10^	3	1.078 × 10^10^	5.3	0.0705
Pure error	8.133 × 10^9^	4	2.03 × 10^9^	-	-
Total	1.37 × 10^14^	16	-	-	-

^a^ SS, sum of squares; ^b^ DF, degree of freedom; ^c^ MS, mean square.

**Table 4:** Significance of the regression coefficients.

Factor	CE ^a^	SE ^b^	*p*-Value
Intercept	7.3 × 10^6^	3.42 × 10^4^	<0.0001
*X*_1_	−6.505 × 10^5^	2.84 × 10^4^	0.0003
*X*_2_	5.633 × 10^5^	5.35 × 10^4^	0.0016
*X*_3_	7.764 × 10^5^	3.62 × 10^4^	0.0044
*X*_1_*X*_2_	5.75 × 10^5^	6.74 × 10^4^	0.007
*X*_1_*X*_3_	8.154 × 10^5^	4.56 × 10^4^	0.042
*X*_2_*X*_3_	8.14 × 10^5^	8.08 × 10^4^	0.034
*X*_1_^2^	−2.668 × 10^6^	3.705 × 10^4^	<0.0001
*X*_2_^2^	−1.144 × 10^7^	1.19 × 10^5^	<0.0001
*X*_3_^2^	−3.98 × 10^6^	5.37 × 10^4^	<0.0001

^a^ CE, coefficient estimate; ^b^ SE, standard error.

Using the optimal formulation (0.64% glycine, 1.02% dl-threonine, and 0.05% Tween 80) determined by the BBD design, an average efficiency of 6.3 × 10^6^ transformants/μg of DNA, which was close to the predicted value (7.38 × 10^6^ transformants/μg of DNA), was archived. These results indicated that RSM is a suitable tool for optimizing the electroporation conditions and provided the first demonstration of using a response surface design to improve the electroporation efficiency in *B. subtilis*.

In contrast with the many experimental designs for wall-weakening agents treatment [14,15,22,32], where the monofactorial experimental designs were used to optimize the concentration of weakening agent and small increases (less than one order of magnitude) in electroporation efficiency were obtained, our results indicated that weakening agents can yield higher increase (two orders of magnitude) with a multifactorial experimental design (RSM).

### 2.6. Effect of Electroporation Buffer on the Electroporation Efficiency

The composition of the electroporation buffer is closely related to the electroporation efficiency. Under the above-described optimized conditions, five types of buffers (MSG, MKK, SMKK, TSM, and TSMKK) were investigated. The first four buffers have been reported as the electroporation buffers for preparing electro-competent in *Bacillus* [9,14,35]. The results, which are listed in Table 5, show that the electroporation buffers supplemented with trehalose have positive effect on the electroporation efficiency and indicate that the TSMMKK buffer is a better electroporation buffer than other tested buffers for *B. subtilis* ZK. In contrast with results of other wild-type *B. subtilis* [20], where an electroporation buffer supplemented with trehalose (TSM) did not affect the electroporation efficiency, our results indicated that trehalose buffer is suitable for the wild-type *B. subtilis* ZK. The similar positive effects have been found in the *B. subtilis* DB104 and *B. subtilis* WB600 [20]. To the best of our knowledge, this is first study applying the TSMMKK buffer that found improved electroporation efficiency.

**Table 5:** Effects of electroporation buffers on the electroporation efficiency.

Buffer	EE ^a^	Reference
MSG	2.86 ± 0.21 × 10^5^	[9]
MKK	1.02 ± 0.19 × 10^5^	[11]
SMKK	4.73 ± 0.41 × 10^5^	[14]
TSM	6.3 ± 0.17 × 10^6^	[35]
TSMMKK	7.4 ± 0.31 × 10^6^	This study

When the OD_600_ value reached 0.5, 0.64% Glycine, 1.02% dl-threonine, and 0.05% Tween 80 were added to the growth medium (LBSP). After shaking for 1 h, the electro-competent cells were prepared. Then, one hundred nanograms of pHT43 plasmid were used for each electroporation experiment. The field strength was 20 kV·cm^−1^. ^a^ EE, electroporation efficiency (cfu/μg of DNA).

In the present study, we found that freezing did not obviously decrease the electroporation efficiency of electro-competent cells within one month (data not shown). Since we did not add any glycerol or other cryoprotectants to store electro-competent cells at −80 °C, we suggest that the trehalose may act as cryoprotectants for electro-competent cells stored at −80 °C. Therefore, the TSMMKK buffer is a good option for improving electroporation efficiency and using the electro-competent cells within the following month.

### 2.7. The Effect of Heat Shock and Plasmid Quantity on the Electroporation Efficiency

**Figure 4:**
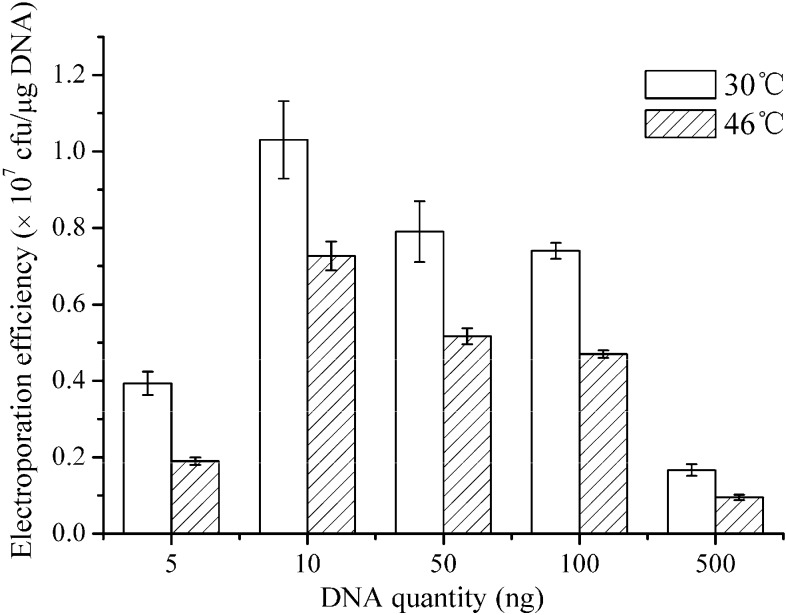
Plots of the electroporation efficiency as a function of the DNA quantity. When the OD_600_ value reached 0.5, the weakening agents (0.64% Glycine, 1.02% dl-threonine, and 0.05% Tween 80) were added to the LBSP medium and the electro-competent competent cells were prepared. *B. subtilis* ZK cells were transformed with various quantities of the plasmid DNA (5–500 ng). The field strength was 20 kV·cm^−1^ and the electroporation buffer was TSMMKK. After electroporation, the cells were incubated in a water bath at 30 or 46 °C. The electroporation experiments were repeated three times, and the data shown are the means of triplicate experiments. The error bars indicate the standard deviations from the average values.

Restriction-modification system is an obstacle of introducing the plasmid into Gram-positive strains. The heat shock is usually applied to reduce the activity of a host restriction-modification system and to improve transformation efficiency [24,36,37,38]. To determine whether the heat shock would improve the electroporation efficiency, a water bath suitable for Gram-positive strains was performed after electroporation according to previous reports [11,24]. In addition, different quantities of the plasmids (5–500 ng) were used to investigate the effect of plasmid quantity on the electroporation efficiency. The results exhibited in Figure 4 show that a water bath at 46 °C decreased the electroporation efficiency of *B. subtilis* ZK and the 10 nanograms yielded higher efficiency than other quantities. In contrast with results of *C. pseudotuberculosis* [22], *C. glutamicum* [24], and *B. amyloliquefaciens* [11], where a water bath at 46 °C after electroporation yielded a significant increase in transformation efficiency, our results indicated that heat treatment did not work for *B. subtilis* ZK and decreased the electroporation efficiency.

### 2.8. Validation of Electroporation Efficiency: A Case Study

Using the optimized conditions (Table 6), the electroporation efficiency was improved to 1.03 × 10^7^ transformants/μg of DNA. To validate the electroporation efficiency, we performed a case study.

**Table 6:** Optimal electroporation condition for *B. subtilis* ZK.

Optimized Factors	Optimal Condition
Growth medium	LBPS
Optical density (600 nm)	0.85
Weakening-agent formulation	0.64% Glycine, 1.02% dl-threonine, 0.05% Tween 80
Time for weakening treatment	1 h
Electroporation buffer	TSMMKK
Plasmid amount	10 ng
Electric field	20 kV·cm^−1^
Time constant	4 ms
Recovery medium	LBMS
Water bath	A water bath at 30 °C for 5 min
Recovery time	3 h

**Figure 5:**
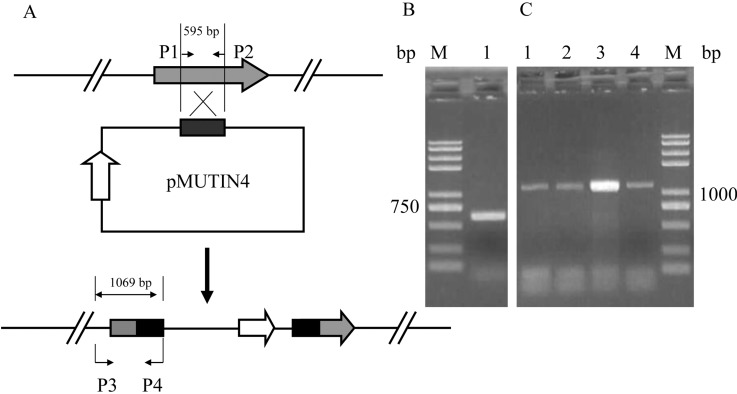
Inactivation of *rapC* gene by gene insertional mutation. (**A**) Construction of insertional mutation of the *rapC* gene; (**B**) Lane **1**: middle encoding region of *rapC* (595 bp) with the P1 and P2 as the primers (**C**) Verification of the single crossover mutants by PCR. Lane **1**–**4**: fragments (1069 bp) with the P3 and P4 as the primers; M: marker.

The suicide pMUTIN4 was used to obtain insertional mutation via a single crossing-event in *B. subtilis* [39]. We used the pMUTIN4 to inactivate a gene (*rapC*). The *rapC* regulates the transcription of *srfA* [40] to influence the expression of lipopeptide antibiotics gene (surfactin) [41]. The gene may be involved in regulation of iturin A biosynthesis. A middle fragment of phosphatase gene (*rapC*) was cloned to pMUTIN4 plasmid and the recombinant plasmid was used to inactivate the *rapC*. Seventy nine transformants were obtained, four transformants of which were randomly selected and verified by PCR. As shown in Figure 5, the mutants have 1069 bp fragment with the P3 and P4 as the primers, indicating that *rapC* genes were inserted by the pMUTIN4 and that the optimized method provided enough efficiency for further genetics studies.

**Figure 6:**
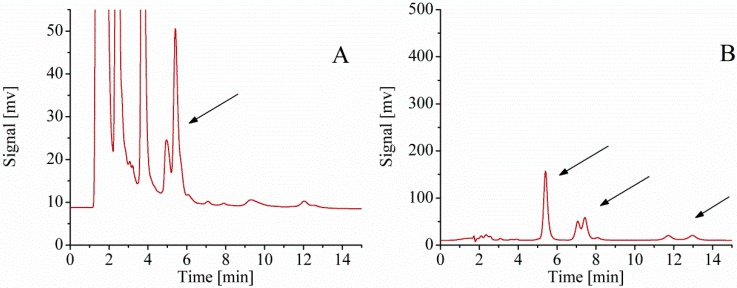

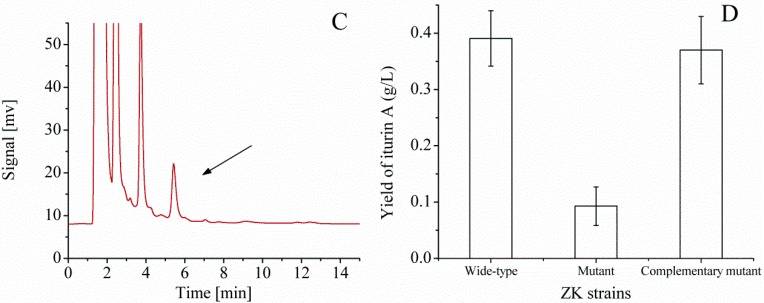
HPLC analysis of the fermentation of wild-type strain and mutant. (**A**) Absorption peak of the iturin A of the wild-type *B. subtilis* ZK; (**B**) Absorption peaks of an iturin A standard sample; (**C**) Absorption peak of the iturin A of the mutant; and (**D**) The yields of iturin A of wild-type strain, mutant, and complementary mutant. The complementary mutant was grown in LB medium in presence of IPTG (0.3 mM).

When compared to the wild-type *B. subtilis* ZK, the *rapC* mutants exhibited markedly decrease (more than half) in iturin A production by shake flask fermentation in LB medium (Figure 6), suggesting that the *rapC* gene was closely related to the iturin A biosynthesis. This study provides the first demonstration of relationship between *rapC* and iturin A. However, further studies were required to confirm the relationship between *rapC* and iturin A production.

## 3. Experimental Section

### 3.1. Chemicals

All of chemicals used in this study were purchased from Sangon Biotech (Shanghai, China), Tiangen (Beijing, China) or cdboruike (Chengdu, China).

### 3.2. Preparation of Desalted Plasmid for Electroporation

*Escherichia coli* JM110 carrying pHT43 (8057 bp; cat; MoBitech GmbH, Goettingen, Germany) was grown in LB (5 g of yeast extract, 5 g of NaCl, and 10 g of tryptone per litre) with 100 μg·mL^−1^ of ampicillin (Amp) at 37 °C, and 180 rpm for 18 h. The pHT43 plasmid was extracted from *E. coli* JM110 using the Plasmid Mini Kit (OMEGA Bio-tek, Norcross, GA, USA) and was desalted as described in the literatures [14,23]. Plasmid quantification was performed using a spectrophotometer (Nanodrop 2000C, Thermo, Wilmington, MA, USA). After purification, the plasmid was dissolved in ddH_2_O and stored at −20 °C.

### 3.3. Screening of the Growth Media for Electro-Competent Cells

A single clone of *B. subtilis* ZK was grown in 3 mL of LB at 30 °C and 150 rpm for 18 h. Then, 2.6 mL of the cultures were respectively added to 40 mL of the growth medium, including LBS (0.5 M sorbitol, 10 g of NaCl, 10 g of tryptone, and 5 g of yeast extract per litre of water), LBSP (0.5 M sorbitol, 0.05 M KH_2_PO_4_, 0.05 M K_2_HPO_4_, 10 g of NaCl, 10 g of tryptone, and 5 g of yeast extract per litre of water), NCM (0.2 M NaCl, 5 g of glucose, 0.1 M K_2_HPO_4_, 5 g of tryptone, 0.5 M sorbitol, 10 mM sodium citrate, 1 g of yeast extract, and 0.2 mM MgSO_4_∙7H_2_O per litre of water), and BHIS (91.1 g of sorbitol, 34 g of Brain Heart Infusion (Difco, Detroit, MI, USA) per litre of water).

### 3.4. The Individual Treatment of Wall-Weakening Agents

A 20% (*w*/*v*) glycine solution and a 5% Tween 80 (*w*/*v*) solution were individually sterilized at 115 °C for 20 min in advance, and 12.5% dl-threonine solution (*w*/*v*) and 50 mg/mL ampicillin were filtered for sterilization. *B. subtilis* ZK was grown in 40 mL of growth medium. When the optical density at 600 nm (OD_600_), as monitored using an U-1800 spectrophotometer (Hitachi, Tokyo, Japan) reached 0.5, the glycine, Tween 80, ampicillin, and dl-threonine solutions were added to the growth medium. After shaking at 37 °C and 200 rpm for 1 h, the electro-competent cells were prepared.

### 3.5. A Combinatorial Treatment of Wall-Weakening Agents

To optimize the concentrations of *X_1_* (glycine), *X_2_* (dl-threonine), and *X_3_* (Tween 80) on the electroporation efficiency (*Y*), a Box-Behnken response surface design (BBD) were performed. A three-variable three-level design was employed, and the values of the variables were selected according to the independent wall-weakening treatment. The coded and un-coded values of the variables are listed in Table 7. The relationship between the three variables and the electroporation efficiency is expressed by the quadratic polynomial shown in Equation (2):

(2) $$Y={\text{β}}_{0}+\sum_{i=1}^{m}{\text{β}}_{i}X_{i}+\sum_{i=1}^{m}{\text{β}}_{i}X_{i}^{2}+\sum_{i<j}{\text{β}}_{ij}X_{i}X_{j}$$

where *Y* is the predicted response (transformants/μg of DNA). *X_i_* and *X_j_* are variables. β_0_, β*_i_*, β*_j_*, and β*_ij_* are constant, linear, square, and interaction coefficients respectively, and m is the number of variable; The BBD design, analysis of variance (ANOVA) and coefficients of the equation were calculated using the Design expert 8.05b software (Stat-Ease Inc., Minneapolis, MN, USA).

**Table 7:** Coded and un-coded values of the variables.

Variables (%)	Un-Coded	Coded
Gly concentration	0.55	−1
	0.65	0
	0.75	1
dl-Thr concentration	0.6	−1
	1	0
	1.5	1
Tween 80 concentration	0.02	−1
	0.06	0
	0.1	1

### 3.6. Screening of the Electroporation Buffer and Preparation of Electro**-**Competent Cells

The cells in growth medium were chilled on ice for 10 min and harvested by centrifugation at 10,000× *g* and 4 °C for 5 min. The cells were purified four times using different buffers, including ice-cold MSG buffer (0.5 M sorbitol, 0.5 M mannitol and 10% glycerol), MKK buffer (0.5 mM MgCl_2_, 0.25 mM K_2_HPO_4_ and 0.25 mM KH_2_PO_4_ per litre of water, pH 7.2), SMKK buffer (272 mM sucrose, 0.5 mM MgCl_2_, 0.5 mM K_2_HPO_4_ and 0.5 mM KH_2_PO_4_ per litre of water, pH7.2), TSM buffer (0.5 M trehalose, 0.5 M sorbitol, and 0.5 M mannitol per litre of water), and TSMMKK buffer (0.5 M trehalose, 0.5 M sorbitol, 0.5 M mannitol, 0.5 mM MgCl_2_, 0.5 mM K_2_HPO_4_ and 0.5 mM KH_2_PO_4_ per litre of water, pH 7.2). After suspension in 1 mL of electroporation buffer, the cells were frozen in liquid nitrogen and stored at −80 °C for later use.

### 3.7. Electroporation

One aliquot (60 μL) of electro-competent cells was thawed on ice and then mixed with the plasmid. After incubation on ice for 3 min, the mixture was loaded into a prechilled electroporation cuvette (0.1-cm electrode gap) and exposed to a single pulse generated by a Gene Pulser System (Bio-Rad, Hercules, CA, USA). After the electrical pulse, 1 mL of LBMS (0.5 M sorbitol, 10 g of NaCl, 10 g of tryptone, 5 g of yeast extract, 0.5 M mannitol, and 0.38 M sorbitol per litre of water) was added immediately to the mixture. After shaking at 37 °C and 200 rpm for 3 h, the cells were harvested and plated on LB agar plates with 5 μg·mL^−1^ chloramphenicol (Cm). The transformants were counted following overnight incubation at 37 °C without agitation.

### 3.8. Treatment of Heat Inactivation

After electroporation, the cells were incubated at 30 or 46 °C for 6 min as previous report [24].

### 3.9. Construction of Plasmids and Analysis of Mutants

A 595 bp fragment encompassing middle encoding region of *rapC* was amplified with primers P-1 (5'-CCCCGGAATTCGGGCTTCTCGATTATTACGTCAACT-3') (with a *Eco*RI site, underlined) and P-2 (5'-CCCGCGGATCCACGGGATCGTCTGTTTCTTTAGCAT-3') (with a *Bam*HI site, underlined). The fragment was digested with *Eco*RI and *Bam*HI and then cloned to pMUTIN4 plasmid. Recombinant plasmid was extracted from *E. coli* and transferred into *B. subtilis* ZK. The mutants were selected on LB plates containing 0.3 μg·mL^−1^ erythromycin (Em). The total DNA of mutant was extracted using the bacterial DNA Kit (OMEGA Bio-tek, Norcross, GA, USA). The inactivate mutants were verified by PCR with the specific primers P-3 (5'-ATGAATCATCTTGAAACCGGCAGTC-3') within upstream of the *rapC* and P-4 (5'-ATCCACGGGATCGTCTGTTTCTTTA-3') within the downstream of the multiple clone site of pMUTIN4.

A 1146 bp full encoding region of *rapC* was amplified with primers P-5 (5'-ACATCAGCCGTAGGATCCATGAAGAGCGGGTTAATTCCTTCTT-3') (with a *BamH*I site, underlined) and P-6 (5'-TAGGCGGGCTGCCCCGGGTTAGATTTCAATTTCATACAAACCC-3') (with a *Sma*I site, underlined). The fragment was digested with *Bam*HI and *Sma*I and then cloned to pHT01 plasmid. Recombinant plasmid was extracted from *E. coli* and transferred into *rapC* mutant. The transformants were selected on LB plates containing 5 μg·mL^−1^ chloramphenicol (Cm) and 0.3 μg·mL^−1^ erythromycin (Em).

### 3.10. Fermentation and Measurement of Iturin A

The ZK strains were activated in LB plates at 37 °C for 36 h. The activated strains were separately inoculated in 5 mL LB mediums (250 mL Erlenmeyer flask) in a shaker at 30 °C with 150 rpm for 22 h. The seed cultures were cultivated in 40 mL LB medium by 10% amount of inoculum at 30 °C, under 150 rpm for 2 days. The extraction and measurement of iturin A as previously reported [42].

## 4. Conclusions

In conclusion, a detailed and systematic protocol that results in improved electroporation efficiency for wild-type *B. subtilis* ZK is described in this manuscript. Using this new method, the electroporation efficiency for *B. subtilis* ZK is improved to 1 × 10^7^ transformants/μg of DNA, which is highest level found for wild-type *B. subtilis*. This efficiency allows the construction of a directed evolution library and allows the knockout of a gene in wild-type *B. subtilis* ZK. We hope that the proposed protocol can facilitate the molecular genetics study in this economically important strain and may shed light on improvements in other wild-type *B. subtlis* strains. In order to validate the electroporation efficiency, a gene (*rapC*) was successfully inactivated using a suicide plasmid pMUTIN4 with the established electroporation method. Moreover, we found that the *rapC* mutants exhibited markedly decrease in iturin A production, suggesting that the *rapC* gene was involved in the regulation of iturin A production.
